# The role of corporate governance structures in mediating the relationship between external supervision, credit appraisal measurement, capital adequacy, and performance of commercial banks in Nepal

**DOI:** 10.1371/journal.pone.0303926

**Published:** 2024-06-13

**Authors:** Tribhuwan Kumar Bhatt, Wenli Wang, Xinghua Dang, Shahina Qurban Jan

**Affiliations:** School of Economic and Management, Xi’an University of Technology, Shaanxi, Xi’an, China; University of Rome Tor Vergata: Universita degli Studi di Roma Tor Vergata, ITALY

## Abstract

This study investigates the role of corporate governance structures as mediators between external supervision, credit appraisal measurement, capital adequacy, and the performance of commercial banks in Nepal. This research sheds light on the significance of effective corporate governance practices within Nepali commercial banks and how certain governance mechanisms may impact bank performance. A quantitative research design was employed, using data from commercial banks in Nepal for this study. Surveys were utilized to collect quantitative data. Structural equation modeling was used as a primary tool to assess the data. The findings add to existing literature about corporate governance and its effects on bank performance in emerging economies such as Nepal. The study’s findings offer valuable insights into the significance of corporate governance structures, external supervision, credit appraisal measurement systems, and capital adequacy for commercial banks’ performance in Nepal. The research methodology adds value to the existing literature using quantitative data collection methods. Its results may have practical ramifications for banks, regulators, and policymakers, suggesting effective governance practices as essential measures for increasing stability and performance at commercial banks.

## 1 Introduction

Corporate governance is essential in shaping the operational efficiency and financial performance of commercial banks around the world [1]. In the context of Nepal, where the banking sector is evolving rapidly amid various internal and external challenges [2], it is essential to understand the interplay between corporate governance structures, external supervision, credit rating measurement, capital adequacy and performance of banks. External supervision, represented by regulatory supervision and compliance requirements, significantly influences the behavior and performance of commercial banks. Regulators in Nepal, notably the Nepal Rastra Bank (NRB), play a vital role in supervising the banking sector to ensure stability and compliance with prudential regulations [3]. Understanding how corporate governance structures interact with external control mechanisms is essential to assess their effectiveness in promoting good banking practices and mitigating risks.

Organizational structure involves creating hierarchies, processes, and mechanisms to direct corporations’ direction, control, and management. Effective governance practices in the banking sector are of particular significance given their systemic impact and potential ripple effect throughout the economy. Rahman [2] addressed that how corporate governance is important for commercial bank performance in Nepal. Besides, external supervision, measured in terms of gender diversity and educational backgrounds are key point for bank performance; previously it was necessary of having multiple perspectives and expertise available when making decisions to maximize overall bank performance [4].

Kurnaiti [5] explored the importance of credit appraisal measurement and corporate governance in Nepalese commercial banks. Also, effective credit appraisal practices, such as rigorous risk evaluation procedures associate with improving governance structures, and establishing that sound credit appraisal practices contribute to overall corporate governance effectiveness, ultimately improving bank performance [6]. The capital adequacy’s role in corporate governance structures and its effect on bank performance are equally important [7]. Additionally, capital adequacy ratios and the effectiveness of governance mechanisms; higher capital adequacy levels help governance practices that ultimately enhance bank performance [8].

Moreover, effective governance practices enable banks to enhance their risk management frameworks, decision-making processes, and financial performance [9]. Corporate governance has long been recognized as integral to financial institution performance and stability, especially in commercial banks. Governance involves establishing structures, processes and mechanisms that oversee corporations’ direction, control and management [2]. Effective governance practices are especially vital in banking, given their systemic significance and potential effects on the overall economy [10].

There is a significant research gap about how strong corporate governance procedures particularly impact the performance of commercial banks in Nepal, despite the acknowledged importance of these policies in fostering ethical behavior and protecting stakeholder interests [5]. Although a significant amount of literature has examined corporate governance procedures in various contexts, there still needs to be more thorough empirical studies that specifically address Nepal’s distinct financial environment [11]. One of the studies by Pareek [12] highlighted the importance of corporate governance and further exploration of financial sectors such as on commercial banks in Nepal.

Key questions about Nepalese banks’ financial performance remain unaddressed [13]. This research challenge is motivated by the urgent need to close this crucial information gap by performing a thorough and rigorous analysis of the factors influencing corporate governance in Nepalese commercial banks and its influence on financial performance [13]. Addressing this issue is critical not only for Nepal’s banking sector’s long-term growth and stability but also for expanding understanding of corporate governance in emerging nations [14]. In line with, this research objectively addresses the extent to which corporate governance structures influence the relationship between external supervision, credit appraisal practices, capital adequacy and bank performance has not yet been explored, particularly in the context of Nepal. This research aims to fill this gap by analyzing the mediating role of corporate governance mechanisms in the influence of external supervision, credit appraisal measurement and capital adequacy on the performance of commercial banks in Nepal. In doing so, this study aims to provide valuable insights to policymakers, regulators, stakeholders’ interest, banking industry professionals and academic researchers seeking to improve the stability and efficiency of Nepal’s banking sector.

## 2 Literature review

### 2.1 External supervision and performance

Resource dependence theory and agency theory support this assertion by suggesting that diverse boards may bring a greater diversity of skills, knowledge, perspectives, and decision-making tools, leading to improved decision-making processes and greater financial results. Furthermore, empirical data supports this assertion by showing a positive association between external supervision and firm performance.

Researchers discovered that companies with diverse boards, particularly concerning gender diversity, exhibited better financial performance [8]. According to them, diversity brings different perspectives, experiences and expertise, ultimately leading to improved decision-making and firm performance [2].

Pareek [12] meta-analysis synthesized the findings of 140 studies related to external supervision and firm performance, showing a positive relationship between the diversity of boards and financial performance measures such as return on equity or return on assets; diverse boards enhance decision-making processes, promote innovation, and overall increase firm performance.

Abdelbadiec [15] research explored the correlation between board gender diversity and firm financial performance with governance structures using an S&P 1500 company sample, and female board representation on boards tending towards greater diversity tends to produce improved financial outcomes [16]. They discovered a positive correlation between female representation on boards tending toward greater performance [17].

Hussain [18] researched the relationship between external supervision and financial performance for Indonesian publicly listed firms, particularly when measured according to gender or educational background diversity. Meanwhile, Asogwa [19] examination of gender diversity’s effect on bank performance found positive associations between gender-diverse boards and improved financial results—further suggesting that banks with more gender-diverse boards tend to produce better financial outcomes than banks without gender-diverse boards.

Based on this literature, the following hypothesis can be formulated:

H1: External supervision positively impacts the performance of commercial banks in Nepal.

### 2.2 Credit appraisal measurement and performance

Effective credit appraisal measurement is critical for commercial banks to assess borrowers and make informed lending decisions accurately. Good practices for credit appraisal measurement, such as rigorous risk evaluation procedures and accurate loan pricing, contribute to building high-quality loan portfolios with reduced credit risk exposure. Recent empirical studies have established a positive relationship between credit appraisal measurement and bank performance.

Credit appraisal measurement and firm performance are integral to commercial bank operations [15]. Credit appraisal involves assessing potential borrowers’ creditworthiness and associated risks before awarding loans or providing credit facilities [2]. Effective credit appraisal practices are vital to building and managing a high-quality loan portfolio, mitigating risk and protecting commercial banks’ financial health. Studies on credit appraisal measurements and firm performance in banking sectors have explored their relationship, with particular attention paid to various dimensions such as risk evaluation, loan evaluation procedures, loan pricing strategies, and the overall effectiveness of credit management [12].

Irawati [16] study examined the impact of credit appraisal quality on bank performance in Nigerian banks. Their results suggested a positive relationship between quality credit appraisal and bank performance. Banks with robust credit appraisal processes, such as comprehensive risk evaluation and accurate loan pricing, exhibited improved financial results. Velte [20] conducted a study analyzing the relationship between credit risk management practices such as credit appraisal measurement and bank performance in the Indonesian banking sector. Their researchers observed a strong positive correlation between effective credit risk management practices, such as credit appraisal measurement, and bank performance indicators, such as return on assets and equity [21].

Feng [22] research explored the effect of credit risk assessment and credit scoring on bank performance in Lebanon. Findings revealed that banks utilizing more advanced credit appraisal techniques, including scoring models, had higher financial performances than banks relying solely on subjective judgment for credit assessment [2]. Studies provide empirical evidence supporting a positive relationship between credit appraisal measurements and bank performance [21]. Effective credit appraisal practices allow commercial banks to identify and manage credit risks, leading to improved loan portfolio health, lower default rates and enhanced financial outcomes for commercial banks.

The following hypothesis can be proposed:

H2: Credit appraisal measurement positively influences the performance of commercial banks in Nepal.

### 2.3 Capital adequacy and performance

Accumulating sufficient capital buffers enables banks to absorb losses, comply with regulatory requirements, and remain financially sound [22]. The theoretical foundation for this relationship can be traced to the capital adequacy framework outlined in the Basel Accords, which emphasizes its role in protecting banks from financial risks. Capital Adequacy and Firm Performance is an integral aspect of banking industry operations. Capital adequacy refers to whether a bank’s capital can absorb potential losses while maintaining financial stability [23]. Adequate capital levels provide safety for bank operations by cushioning potential unexpected losses and safeguarding depositors and stakeholders [24].

Numerous studies have investigated the relationship between capital adequacy and firm performance, especially within banking, using measures such as capital adequacy ratios defined by regulatory frameworks like Basel Accords. Feng [22] research focused on Islamic and conventional banks in the Middle East with different capital adequacy ratios in place; their findings indicated a positive association between high capital adequacy ratios and bank outcomes [10].

Pareek [12] conducted a study to analyze the relationship between capital adequacy and bank profitability among European banks, with their research showing a strong positive relationship. Furthermore, Chakroun [25] investigated this same topic by looking at capital adequacy’s relationship to bank risk-taking behavior in Tunisia’s banking sector—showing that those with higher capital adequacy ratios displayed reduced levels of risk-taking behaviors leading to better risk management practices that ultimately contributed to better financial results overall.

Therefore, the following hypothesis can be formulated:

H3: Capital adequacy positively affects the performance of commercial banks in Nepal.

### 2.4 Corporate governance structures and performance

Corporate governance structures refer to the mechanisms, processes and practices through which corporations are directed, controlled and managed. This may include board membership composition and the presence of independent directors as well as audit committees, risk management frameworks and internal controls [23]. Effective governance structures are expected to increase transparency, accountability, risk management, and decision-making processes, ultimately improving firm performance [8]. Corporate governance plays a pivotal role in shaping the performance success of corporations [8].

The composition of a board of directors is an integral element of corporate governance structures. According to research, having an inclusive and diverse board may result in improved decision-making and firm performance—for instance, Hussain [18] found a correlation between external supervision as measured by gender diversity and firm performance in the UK context. Independent directors are essential in assuring board independence from management while simultaneously representing shareholder interests. Studies have demonstrated their importance by associating their presence with improved firm performance. Rahman [2] research found a positive relationship between the proportion of independent board directors and firm value.

Audit committees oversee an organization’s financial reporting, internal controls, and risk management processes. Effective audit committees have been linked with higher firm performance; Dat [9] conducted research that showed their presence positively impacted firm performance in terms of Tobin’s Q within US contexts. Strong risk management frameworks are crucial in identifying, assessing and mitigating company risks. Implementing effective risk management structures has been linked with better firm performance; Obaje [26] conducted research investigating this relationship among Spanish firms—finding a positive correlation between risk management and firm value.

Strong internal control systems help ensure compliance with laws and regulations, safeguard assets and increase the reliability of financial reporting. Studies have demonstrated the positive effect effective internal controls have on firm performance; for example, Tiep Le [6] discovered a correlation between the quality of internal controls and firm performance in Australia; empirical studies support this correlation; thus, it is safe to propose this hypothesis:

H4: Strong corporate governance structures positively impact the performance of commercial banks in Nepal.

### 2.5 Mediation of corporate governance structures between external supervision and financial performance

Studies suggest corporate governance structures are intermediaries between external supervision and firm financial performance. Effective governance mechanisms can leverage the benefits of external supervision for increased decision-making, risk management, and overall financial performance [21]. Studies on corporate governance structures have focused on their role as mediators between external supervision and financial performance. One such study by Abdelbadie [15] explored the mediating effect of board independence on the relationship between external supervision (gender diversity) and firm financial performance; results indicated that board independence partially mediated this relation, suggesting the presence of independent directors may have altered it [16].

The study Endrikat [10] explored the mediating role of board independence and control mechanisms (such as CEO duality and ownership concentration) on the relationship between board racial diversity and firm financial performance. Their results suggested that board independence partially mediated this relation, suggesting that its influence on firm financial performance was at least partly mediated by this aspect of governance structure independence.

These studies demonstrate the significance of certain governance structures, such as board independence, as mediating factors between external supervision and firm financial performance [27]. Governance mechanisms may enhance or dampen its effects by altering organizational decision-making processes and monitoring procedures [28]. Hence these studies indicate the power of corporate governance structures—such as independent directors and control mechanisms in mediating this relationship between external supervision and firm financial performance.

Thus, the following hypothesis can be formulated:

H5: Corporate governance structures mediate the relationship between external supervision and the financial performance of commercial banks in Nepal.

### 2.6 Mediation of corporate governance structures between credit appraisal measurement and financial performance

Corporate governance structures can facilitate equilibrium between credit appraisal measurement and bank financial performance. Robust governance practices facilitate the implementation of effective credit appraisal processes, guaranteeing their accuracy, reliability, and consistency [29]. Studies have explored the mediating role that corporate governance structures have between credit appraisal measures and firm financial performance. However, it is widely recognized that effective governance structures can substantially affect credit appraisal processes and their associated effects on financial performance [30]. Corporate governance mechanisms, including independent directors, strong risk management frameworks and transparent reporting practices, play an essential part in credit appraisal. These measures ensure credit decisions are based on objective criteria such as adequate risk analysis and sound governance practices.

Kartsonakis [31] conducted an in-depth analysis of how board governance impacts credit ratings within banking institutions. Findings indicated that strong governance, including independence and expertise of board members, was associated with higher credit ratings. Governance structures impact credit appraisal processes and, thus, firms’ access to credit and overall financial performance. Research by Okera [32] explored the relationship between capital adequacy and risk-taking behavior in the Tunisian banking sector, which closely relates to credit appraisal practices. This study concluded that capital adequacy as part of corporate governance structures influenced bankers’ risk-taking behavior [20]. The paper indicates that governance structures involving credit appraisal measurement as part of risk management indirectly determine the relationship between credit appraisal measurement and firm financial performance.

Therefore, the following hypothesis can be proposed:

H6: Corporate governance structures mediate the relationship between credit appraisal measurement and the financial performance of commercial banks in Nepal.

### 2.7 Mediation of corporate governance structures between capital adequacy and financial performance

Corporate governance structures also mediate between capital adequacy and bank financial performance [33]. Strong governance mechanisms, including risk management frameworks and internal controls, are critical to maintaining optimal capital levels while optimizing the allocation and utilization of resources [30]. The mediating role of corporate governance structures between capital adequacy and firm financial performance refers to how governance practices influence the relationship between capital adequacy and firm financial performance, acting as a mediator by shaping capital adequacy’s impact on firm performance [31].

Limited research has specifically explored the mediatory role of corporate governance structures between capital adequacy and firm financial performance, yet it is widely recognized that effective governance structures can significantly alter this relationship [34]. Therefore, effective mechanisms like board composition, risk management frameworks and internal controls all play an essential part in assuring capital adequacy regarding firm performance [30].

Kartsonakis [31] research investigated the relationship between capital adequacy and bank financial performance, specifically examining how corporate governance plays out within banking. Findings indicated that strong corporate governance practices, including independent board oversight and risk management mechanisms, mediated capital adequacy and bank financial performance [35]. Governance structures appear to influence how effectively capital adequacy drives a firm financial performance, according to research conducted by [36]. Their study explored the influence of corporate governance on capital adequacy levels and bank risk-taking behavior.

Thus, the following hypothesis can be formulated:

H7: Corporate governance structures mediate the relationship between capital adequacy and the financial performance of commercial banks in Nepal.

**Fig 1:**
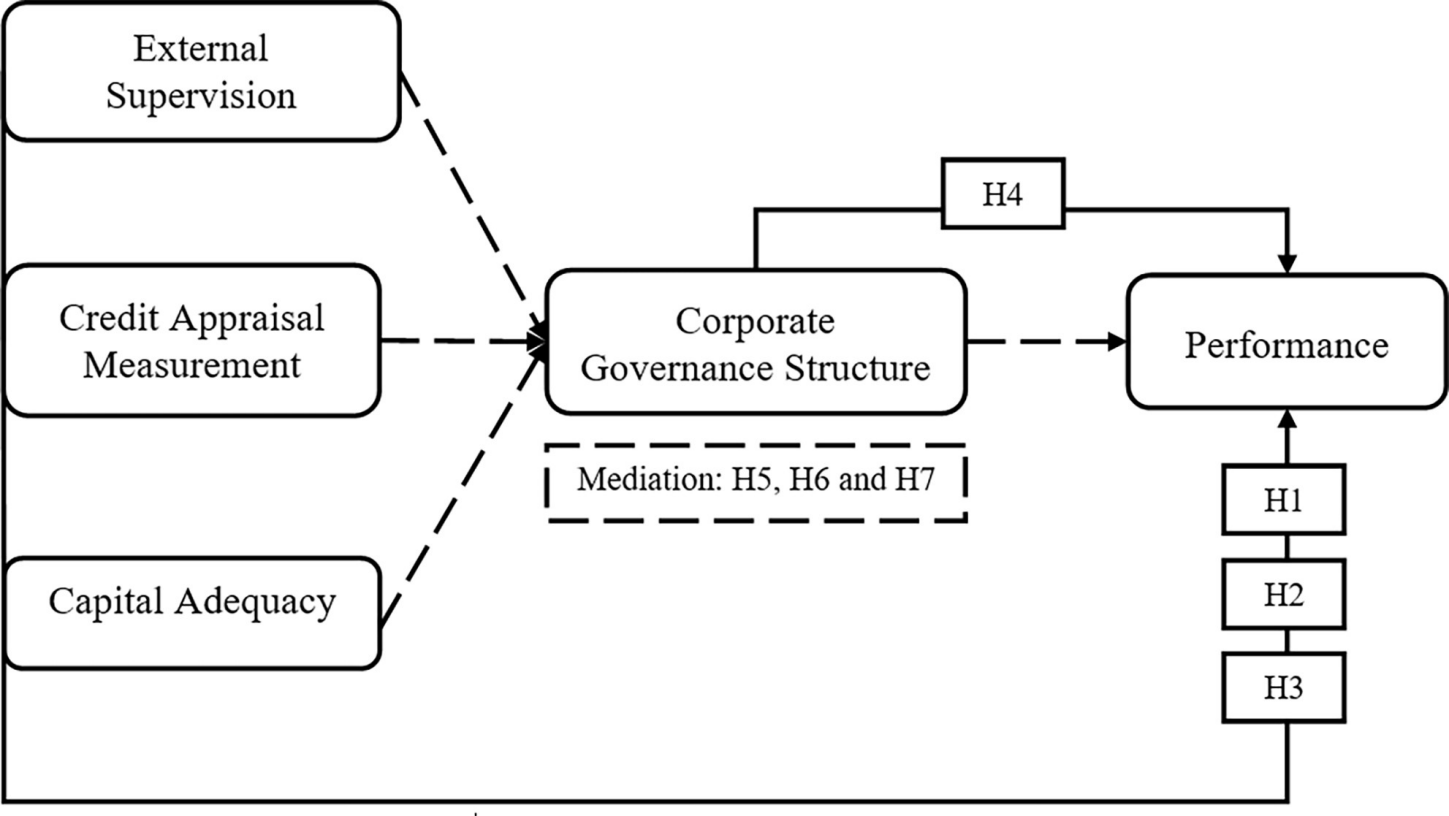
Theoretical framework.

According to Agency Theory, external supervision, often in the form of regulatory oversight and governance mechanisms, mitigates agency problems in banks. The literature provides mixed findings regarding the impact of external supervision on bank performance. Some studies [37] have shown a positive relationship, while others [38] found no significant impact. Our hypothesis aims to clarify this relationship in the context of Nepal Commercial Bank. The relationship between capital adequacy and performance aligns with Resource Dependency Theory. Maintaining an adequate capital base provides banks with the resources needed for growth and financial stability. This hypothesis is supported by previous studies [39, 40]. In the current research, overall theoretical framework builds the relationships (direct and indirect) with the bank performance as shown in Fig 1.

## 3 Research method

Nepal’s commercial banks are crucial to the nation’s financial industry and economy as a whole [41]. These organizations serve as crucial intermediaries, easing the transfer of money between savers and borrowers. Commercial banks provide various financial services, such as deposit, lending, and investing operations, that support economic growth and development [30]. Commercial banks in Nepal offer consumers, businesses, and governmental organizations necessary financial services. They provide numerous financial needs-satisfying solutions, including loans, investment options, and savings accounts. Commercial banks are also essential in mobilizing public savings and directing them toward profitable initiatives, which boost economic activity. Additionally, Nepal’s GDP (gross domestic product) would not be the same without the substantial contribution of commercial banks. Interest, fees, and commissions are generated by its operations, and these earnings eventually go toward the nation’s total economic production. Commercial banks, as major participants in the financial system, are essential to the stability and growth of Nepal’s economy [42].

Based on the Cochran [43] formula, this technique is well-recognized for minimizing sampling deviation and computing a valid sample size. Employing the Cochran formula, a suitable sample was measured and estimated for current research.

z = z score

α = margin of error

$\hat{p}$ = population proportion

$n=\frac{z^{2}\times \hat{p}(1-\hat{p})}{\alpha^{2}}=\frac{1.96^{2}\times 0.2(1-0.2)}{0.05^{2}}$ = 385, therefore, n = 410 was considered for study.

A sample size covering 410 representatives from active commercial banks in Nepal was selected in order to fulfil the research objective. To gather data from such institutions, scholar used a questionnaire survey. It is highly recommended that the study focus on collecting responses from key informants, who are believed to be familiar respondents proficient in catering truthful replies [44]. It is broadly accepted that simple randomization of the sample helps scholars identify the sample to target the population [45]; hence, in order to reach the targeted population, this technique was used.

In Table 1, the sample contained 410 participants, and no missing values were found in the data. All 410 responses were acknowledged in this research. Three hundred twenty-two males, or 79% of the participants overall, represented the sample, whereas eighty-eight females comprised 21%. The respondents characterized 25%, 46%, 19%, and 10% of the selected sample, respectively, and according to participants, age years-wise ranged from 20 to 30 (102), 31 to 40 years (189), 41 to 50 years (76), and 51 years or older (43).

**Table 1:** Demographics statistics.

Demographics	Frequency	Percentage
**Gender**		
Male	322	79%
Female	88	21%
Total	410	100%
**Age group**		
Between 20 to 30	102	25%
Between 31 to 40	189	46%
Between 41 to 50	76	19%
51 or higher	43	10%
Total	410	100%
**Managerial Designations**		
Top	111	27%
Middle	192	47%
Lower	107	26%
Total	410	100%
**Educational Background**		
Associate Finance Certification	129	32%
Bachelor	178	43%
Master or higher	103	25%
Total	410	100%
**Experience**		
1 to 5 years	122	30%
6 to 10 years	174	42%
above 10	114	28%
Total	410	100%

Regarding managerial designations, the research involved 111 participants (27%) at the top level, 192 participants (47%) at the middle level, and 107 participants (26%) at the lower level. Concerning educational qualifications, there were 129 participants (31.5%) with associate certifications, 178 participants (43.4%) with bachelor’s degrees, and 103 participants (25.1%) with master’s degrees or higher.

Following a categorization of experience, the participants had diverse levels of exposure, with 122 participants (30%) having 1 to 5 year(s) of experience, 174 participants (42%) having six-to-ten years of experience, 114 participants (28%) having eleven or more years of experience. Table 1 provides a summary of these data.

For measurement scales, see *S1 Appendix*, used in this study varied depending on the variables being assessed. When measuring external supervision, variables like gender diversity, ethnic diversity or educational diversity were considered, the items were adopted from the work of Pearce[46]. Credit appraisal measurement included factors like quality of the credit assessment process, adherence to lending standards or accuracy of risk evaluation, questions were adopted from the work of Chepkorir [47]. Capital adequacy was assessed using established financial ratios such as Capital Adequacy (CA), which compares bank capital with risk-weighted assets, original items were acquired from the study of Olalekan & Adeyinka [48]. Performance was measured through measures like return on assets (ROA), return on equity (ROE), net income or market-based measures like stock prices and market capitalization, items obtained from the research of Re, Schmidt [49]. Corporate Governance scale was adopted form Ho [50]. Data collected through surveys and it was the foundation for analyzing relationships among variables and testing proposed hypotheses.

Before an actual survey, this research has been approved by the Research Ethics Committee of Xian University of Technology. The research methodology involved gathering data through surveys, questionnaire instrument. With particular attention paid to external supervision, credit appraisal measurement, capital adequacy and firm performance variables. Scales were employed for measuring each variable to accurately analyze relationships among them and test hypotheses related to research hypotheses.

## 4 Result and discussion

Using Smart-PLS, initially measurement model consists of with its factor loadings, average variance extracted values and composite reliability ratings of variables as an evaluation method of measurement models [51], has been used to evaluate measurement models. Cronbach’s alpha values represent the accuracy, reliability, consistency and dependability in data; and validity presents factor loadings and average variance extracted values (AVE) is presented in the study.

### 4.1 Measurement model

For each variable in a study, its CR value> 0.7 and AVE must exceed 0.5 to exhibit high relevance and guarantee minimum standards. After this initial stage is completed, various methods should be employed to test for the discriminant validity of data collected during subsequent phases; according to [52], factor loading> 0.6, HTMTs and cross-loadings occur when one factor relies heavily on another than it does other factors. All factor loadings were retained in this study as per standards, factor load minimum was 0.624 and maximum was 0.900, hence achieved standard threshold, as shown in Table 2.

**Table 2:** Confirmatory factor analysis, reliability and validity.

Constructs	Factor Loading				
**Capital Adequacy:** CA = 0.914; rho_A = 0.914; CR = 0.936; AVE = 0.745					
CAD1	0.820				
CAD2	0.854				
CAD3	0.893				
CAD4	0.874				
CAD5	0.874				
**Credit Appraisal Measurement**: CA = 0.943; rho_A = 0.946; CR = 0.952; AVE = 0.688					
CAM1		0.838			
CAM2		0.865			
CAM3		0.816			
CAM4		0.841			
CAM5		0.761			
CAM6		0.846			
CAM7		0.841			
CAM8		0.848			
CAM9		0.804			
**Corporate Governance:** CA = 0.889; rho_A = 0.894; CR = 0.912; AVE = 0.566					
CG1			0.692		
CG2			0.665		
CG3			0.712		
CG4			0.708		
CG5			0.775		
CG6			0.829		
CG7			0.841		
CG8			0.777		
**External Supervision**: CA = 0.951; rho_A = 0.958; CR = 0.960; AVE = 0.772					
ES1				0.773	
ES2				0.893	
ES3				0.886	
ES4				0.899	
ES5				0.881	
ES6				0.912	
ES7				0.900	
**Performance:** CA = 0.931; rho_A = 0.931; CR = 0.943; AVE = 0.625					
PER1					0.624
PER10					0.815
PER2					0.625
PER3					0.789
PER4					0.848
PER5					0.847
PER6					0.849
PER7					0.830
PER8					0.798
PER9					0.832

#### 4.1.1 Reliability and validity

Standardized reliability and validity indorsed by [53] that thresholds for reliability and validity as this research has followed the same. External supervision exhibits excellent internal consistency reliability as indicated by Cronbach’s alpha of 0.951, high reliability with a rho_A of 0.958 and satisfactory reliability with CR of 0.960. An average variance accounted for by this construct (AVE = 0.772) is also evident; thus, 77.2% of the variance is accounted for by itself. Credit Appraisal Measurement also exhibits high internal consistency reliability with an alpha value of 0.943. The construct offers reliable measurement with a rho_A of 0.946 and satisfactory reliability with CR of 0.952; an AVE = 0.688 indicates that 68.80% variance is due to this construct itself. Capital Adequacy exhibits outstanding internal consistency reliability with a Cronbach’s Alpha value of 0.914, showing reliable measurement with rho_A of 0.914 and satisfactory reliability with CR at 0.936. Capital adequacy has an Average Variance Explained (AVE) value of 0.745, indicating that their construct explains 74.50% of the variance observed. Cronbach’s Alpha of 0.889 indicates reliable internal consistency reliability within corporate Governance structures. This construct exhibits high reliability with a rho_A of 0.894 and satisfactory reliability with a CR of 0.912. An average variance estimate (AVE) of 0.566indicates that this construct can explain 56.60% of variance observed indicators. Performance has excellent internal consistency reliability with Cronbach’s alpha of 0.931. Additionally, its construct has high reliability with a rho_A of 0.931 and satisfactory reliability with CR of 0.943; an AVE score of 0.625 indicates that approximately 62.50% of variance observed indicators can be explained by its construct, Table 2 highlights Cronbach’s alpha, composite reliability and average variance explained values.

#### 4.1.2 Fornell-Lacker criterion

The Fornell-Lacker Criterion assesses discriminant validity among constructs by comparing square roots of average variance extracted (AVE) with correlations among them.

The Average Variance Estimator for capital adequacy stands at 0.863, meaning that this construct explains 86.3% of variance observed indicators related to external supervision. Credit Appraisal Measurement also has an Average Variance Estimator value of 0.829. This result indicates that 82.9% of the variance observed indicators for Credit Appraisal Measurement can be attributed directly to its construct. Corporate governance structures Volatility Envelope has an AVE value of 0.752. The results show that its construct explains 75.2% of the variance observed in indicators related to corporate governance structures; thus, External supervision account for 0.879 of this variance. The research indicates that their construction can explain 87.9% of external supervision indicators variance. Furthermore, Performance’s AVE stands at 0.790; thus, suggesting that its framework can explain 79.0% of the variance.

Table 3 illustrates the discriminant validity using Fornell-Larcker criterion suggested by [53], the correlation between Capital Adequacy and Credit Appraisal Measurement is 0.531; Corporate Governance with Capital Adequacy stands at 0.594, while that between External supervision and Capital Adequacy stands at 0.444. Correlation Analysis shows a correlation of 0.505 between Capital Adequacy and Performance, Credit Appraisal Measurement with Corporate Governance Structures of 0.816, and External Supervision at 0.441. Credit Appraisal Measurement and Performance have an approximate correlation value of 0.486; Corporate Governance Structures with External Supervision and performance has 0.558 and 0.591 correlation, while External Supervision and performance stands correlation of 0.480. These correlation values provide valuable insight into their relationships.

**Table 3:** Fornell-Larcker criterion.

Constructs	CAD	CAM	CGS	ES	PER
Capital adequacy	0.863				
Credit appraisal measurement	0.531	0.829			
Corporate governance structures	0.594	0.816	0.752		
External supervision	0.444	0.441	0.558	0.879	
Performance	0.505	0.486	0.591	0.480	0.790

#### 4.1.3 Heterotrait-Monotrait (HTMT)

Another method to assess the discriminant validity is Heterotrait-Monotrait (HTMT), a value must be less than 0.90 see Table 4. The relational values explain that capital adequacy exhibits a moderate relationship with Credit Appraisal Measurement (0.569), suggesting some relationship between capital adequacy and credit appraisal measurement in an organization. Furthermore, Capital adequacy strongly correlates with corporate governance structures (0.665). This shows a clear association between capital adequacy and corporate governance structures levels within an organization and a moderate correlation with external supervision (0.475). Capital Adequacy has a moderate relationship with performance (0.543). This suggests a moderate link between capital adequacy and overall governance structures in place and the performance of an organization.

**Table 4:** Heterotrait-Monotrait.

Constructs	CAD	CAM	CGS	ES	PER
Capital adequacy					
Credit appraisal measurement	0.569				
Corporate governance structures	0.665	0.869			
External supervision	0.475	0.455	0.604		
Performance	0.543	0.514	0.650	0.499	

Credit Appraisal Measurement has a strong correlation with Corporate Governance Structures (0.869), suggesting a strong relationship between Credit Appraisal Measurement and levels of Corporate Governance Structures in an organization. On the other hand, Credit Appraisal Measurement displays good relationships with External Supervision (0.455) and Performance (0.514). These strong associations between credit appraisal measurement and overall organizational performance highlight its relevance for effective credit management practices.

Corporate Governance Structures demonstrates a good relationship with External Supervision (0.604), suggesting there may be good correlation between Corporate Governance Structures levels and those implemented within an organization’s supervision level. On the contrary, Corporate Governance Structures good correlates with performance (0.650). This indicates a strong link between Credit Appraisal Measurement and organizational performance, as evidenced by a high and positive HTMT value of 0.869. Furthermore, Credit Appraisal Measurement shows evidence of their performance impacting organizational effectiveness, as demonstrated by this correlation. Consequently, the effectiveness and quality of Credit Appraisal Measurement implemented within an organization immensely influence its performance outcomes. A higher correlation value indicates that well-designed governance structures and appraisal process may positively contribute to improved organizational results.

### 4.2 Structural model

Structural Equation Modeling (SEM) was chosen as the primary analytical tool for this study. SEM is a powerful and flexible statistical technique that enables the simultaneous examination of complex relationships among variables. It goes beyond simple regression analysis by considering both observed and latent (unobserved) variables [53].

In SmartPLS, researchers usually evaluate the screening for their structural equation models (SEM) using a range of cutoff values. R^2^, Q^2^, f^2^, and VIF are important levels to consider when assessing various model components [51]. The coefficient of determination, or R2, shows the percentage of the endogenous construct’s variance that the exogenous constructs account for. Higher values indicate greater model fit. Typically, it is between 0 and 1. R^2^ values were found within the range for this research. Predictive relevance, or Q^2^, assesses how well the model can predict endogenous constructs. When cross-redundancy values must be higher than zero, all exogenous variables were above zero in extant research. The degree to which exogenous and endogenous factors are related is shown by the f^2^ measure of effect size in PLS-SEM. Researchers typically regard f^2^ values greater than 0.02 as indicating a significant impact size; hence, all values exceeded 0.02 in this research. The variance inflation factor, or VIF, evaluates the degree of multicollinearity among exogenous components. It is generally accepted that multicollinearity is not a significant worry in the model when the VIF value is less than 5. The current research indicated no value was greater than five and fell within a standard range, as exhibited in Table 5. In SmartPLS, researchers may guarantee the model relevance of their structural equation models by establishing suitable thresholds for R^2^, Q^2^, f^2^, and VIF.

**Table 5:** Predictive relevance.

Constructs	f^2^	Q^2^	R^2^	VIF
**Capital Adequacy**	0.067	-	-	1.609
**Credit Appraisal Measurement**	1.016	-	-	3.031
**Corporate Governance**	-	0.408	0.733	3.739
**External Supervision**	0.115		-	1.498
**Performance**	-	0.239	0.409	-

Based on the provided information, Table 6 contains coefficients, standard beta values, standard deviations (SD), t-values, p-values, results that have been calculated using Smart-PLS 3.3.9. Table 6 presents the hypotheses results for direct and indirect effects. Hypothesis 1 was accepted since its standard beta value of 0.185 indicates a positive relationship between external supervision and performance, with an associated t-value greater than 1.96 and a significant (p = 0.000) p-value (hence accepting this hypothesis). Hypothesis 2 was rejected in comparison since credit appraisal measurement has not an equally significant positive effect on firm performance. Hypothesis 3 was supported, suggesting a positive relationship between capital adequacy and performance, with an average standard beta value of 0.207, indicating that this relationship is positively correlated. Furthermore, Hypothesis 4 is also supported, showing that corporate governance structures have substantially affected firm performance.

**Table 6:** Structural equation modeling.

S.NO	Hypothesis	Std Beta	SD	T values	P values
H1	External supervision→Performance	0.185	0.047	3.947	0.000
H2	Credit Appraisal measurement→Performance	-0.009	0.076	0.116	0.907
H3	Capital Adequacy→Performance	0.207	0.047	4.433	0.000
H4	Corporate Governance Structure→Performance	0.372	0.081	4.608	0.000
H5	External supervision→ Corporate Governance Structure→Performance	0.076	0.021	3.677	0.000
H6	Credit Appraisal measurement→ Corporate Governance Structure→Performance	0.237	0.053	4.474	0.000
H7	Capital Adequacy→ Corporate Governance Structure→Performance	0.061	0.02	3.023	0.003

The standard beta value of 0.076 indicated a positive relationship between external supervision and corporate governance structures, as evidenced by its t-value exceeding 1.96 and its significant p-value (p = 0.000). Hypothesis 5 was accepted, as its evidence supports the claim that external supervision positively impacts corporate governance structures, which in turn have an indirect positive effect on firm performance. Hypothesis 6 was accepted by its standard beta value being 0.237, signifying a positive relationship between credit appraisal measurement and corporate governance structures, with t-value exceeding 1.96 and significant (p = 0.000) p-values; these results suggest effective credit appraisal measurement positively influences governance structures that in turn have an impact on firm performance.

**Fig 2:**
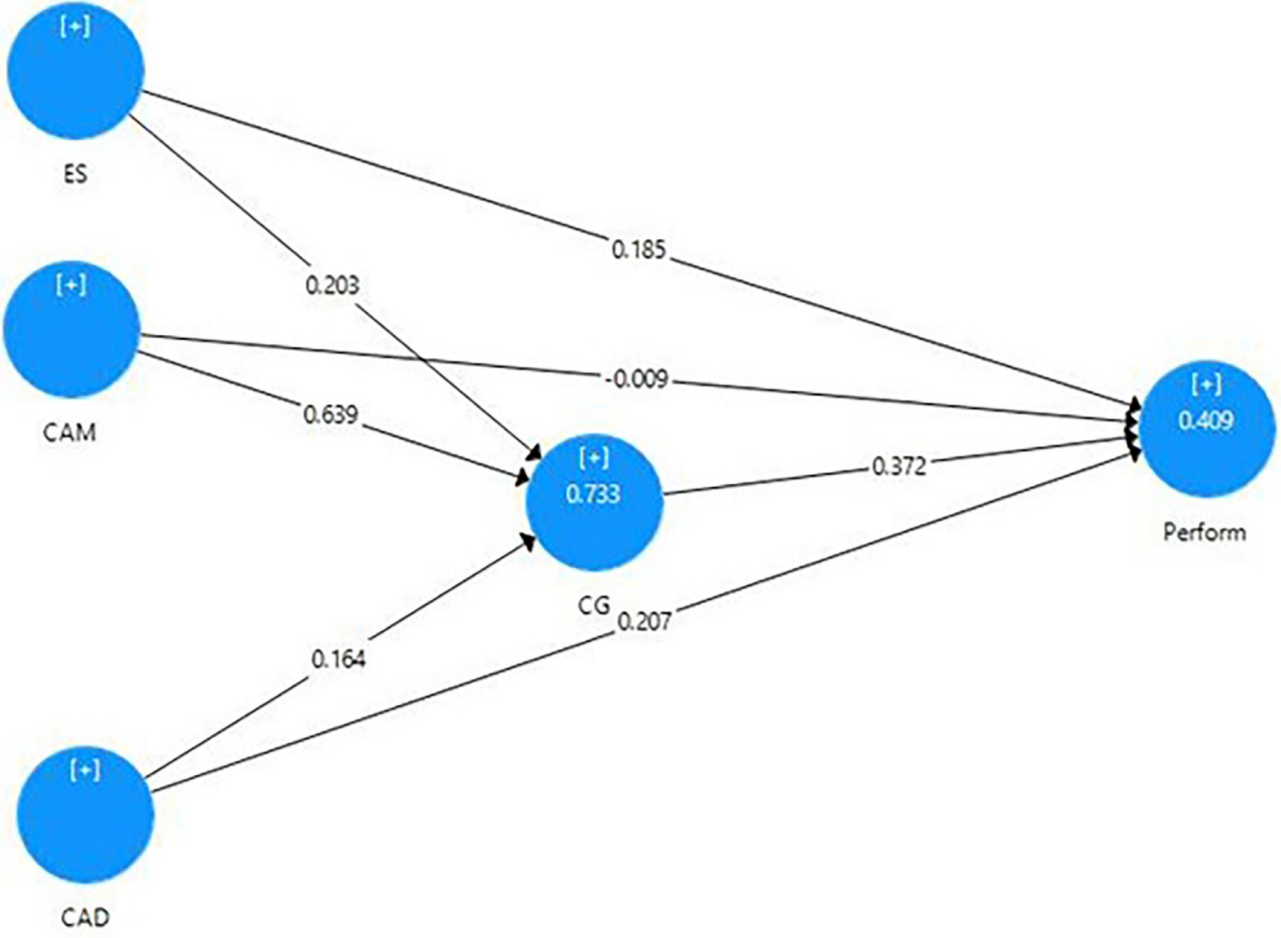
Structural model.

The standard beta value is 0.061, suggesting a positive relationship between capital adequacy and corporate governance structures. Furthermore, its t-value of 3.023 exceeds 1.96, and its p-value (p = 0.003) indicates its significance (reported in Table 5). Hypothesis 7 can therefore be accepted; showing that capital adequacy positively affects corporate governance structures and improves firm performance. Bases on the results, exhibited in Table 6 and Fig 2, indicate that external supervision, capital adequacy and corporate governance structures all play significant positive roles in firm performance in commercial banks. Corporate governance structures’ ability to mediate between external supervision and performance, capital adequacy and performance and corporate governance structures on performance is supported by significant coefficients, t-values, and p-values.

### 4.3. Discussion

This research offers valuable insight into the relationship between external supervision, credit appraisal measurement, capital adequacy, corporate governance structures and firm performance for commercial banks in Nepal. Furthermore, it highlights their significance as drivers of the performance outcomes of banks.

H1: External Supervision → Performance

As hypothesized, the relationship between external supervision and the bank’s performance was found to be significant (Std Beta = 0.185, p = 0.000). This aligns with previous research byBermpei [37] and Chen [38], which similarly identified the positive influence of external supervision on bank performance. The regulatory and external supervision appear to foster a sense of accountability and responsibility among bank stakeholders, which, in turn, enhances performance. These findings underscore the importance of regulatory compliance and the need for a robust external supervisory framework in Nepal Commercial Bank.

H2: Credit Appraisal Measurement → Performance

Our results indicate a non-significant relationship between credit appraisal measurement and the bank’s performance (Std Beta = -0.009, p = 0.907).Although the result is insignificant but literature partially supports the findings of Binuyo [40] and Cregan [54] emphasizing the vital role of robust credit appraisal practices in reducing credit risk and enhancing financial performance. Effective credit appraisal ensures informed lending decisions and risk mitigation, thus contributing to improved performance.

H3: Capital Adequacy → Performance

The results reveal a significant positive association between capital adequacy and the bank’s performance (Std Beta = 0.207, p = 0.000). This aligns with the research conducted by Singh N [39] and Binuyo [40] highlighting the importance of maintaining an adequate capital base. Sufficient capital resources support growth and financial stability, which, in turn, positively impacts bank performance. This emphasizes the need for regular assessments to ensure capital adequacy in line with regulatory requirements.

H4: Corporate Governance Structure → Performance

The result shows a significant positive relationship between corporate governance structure and the bank’s performance (Std Beta = 0.372, p = 0.000). This corresponds with the findings of Flew [55] underlining the importance of effective governance in organizations. Clear roles and responsibilities for board members, transparency in decision-making, and stakeholder alignment within governance structures are critical components. These results accentuate the significance of sound governance practices in Nepal Commercial Bank.

H5: External Supervision → Corporate Governance Structure → Performance

Hypothesis 5 proposed an indirect relationship between external supervision, corporate governance structure, and performance. Our results confirm this relationship (Std Beta = 0.076, p = 0.000). The mediating role of corporate governance structure between external supervision and performance is consistent with Kartsonakis [31], who found that strong governance practices are associated with higher credit ratings. This reinforces the importance of governance in enhancing bank performance, especially in the presence of external supervisory measures.

H6: Credit Appraisal Measurement → Corporate Governance Structure → Performance

Hypothesis 6 also suggested an indirect relationship between credit appraisal measurement, corporate governance structure, and performance, which our results support (Std Beta = 0.237, p = 0.000). This underlines how governance mechanisms mediate the relationship between credit appraisal measures and the bank’s performance, as proposed by Cregan [54]. Effective governance practices ensure credit decisions are based on objective criteria, contributing to improved financial outcomes.

H7: Capital Adequacy → Corporate Governance Structure → Performance

Hypothesis 7 proposed another indirect relationship, between capital adequacy, corporate governance structure, and performance. Our analysis indicates a significant mediating role of corporate governance structures and mechanisms between these factors (Std Beta = 0.061, p = 0.003). These findings are consistent with the research of Kartsonakis [31] and emphasize the paramount importance of sound governance practices in shaping the relationship between capital adequacy and bank performance.

While our results are in line with previous studies, such as those by [37, 39, 40], there are still some specific studies not accounted for in this analysis, and further research is needed to fully understand the nuances of these relationships. Future research could delve into the causal mechanisms behind these associations, considering external factors like regulatory changes, economic conditions, and technological advancements.

Concerning external supervision, analysis shows a positive relationship with firm performance. This result aligns with prior research that suggests diverse boards bring a wider array of skills, knowledge, and perspectives to decision-making processes leading to improved financial results and thus contributing to bank success overall. Commercial banks in Nepal have diverse boards that likely contribute significantly towards overall success, as evidenced by higher profits than banks that lack such boards. Recent studies show that credit appraisal measurement can have an important influence on firm performance.

Accurate measurement of credit risk is integral part for banks when it comes to maintaining the quality of their loan portfolios, and this finding underscores its significance in improving the financial performance of commercial banks. This study highlighted a positive association between capital adequacy and firm performance. Adequate capital levels allow banks to absorb potential losses and maintain financial stability; higher capital adequacy ratios reduce risks and increase investor trust, providing a cushion against risks while building confidence among investors. Results suggested that commercial banks in Nepal with higher capital adequacy levels tend to show improved performance.

Corporate governance structures have also been shown to have an enormously significant effect on firm performance. Well-designed governance mechanisms such as board composition, presence of independent directors, and risk management frameworks contribute to increased transparency, accountability, and risk management practices. This helps positive impact on the performance outcomes of commercial banks. Mediation analysis indicates that corporate governance structures mediate between external supervision and firm performance, credit appraisal measurement and firm performance and capital adequacy and performance—suggesting that effective corporate governance practices play a crucial role in translating benefits such as external supervision, credit appraisal measurement and capital adequacy into improved firm performance.

## 5 Conclusion

Based on the findings of this research, external supervision, credit appraisal measurement (to some extent), capital adequacy levels and corporate governance structures are major influencing factors in Nepalese banks’ performance. Furthermore, diverse boards, effective credit appraisal practices, increased capital adequacy levels, and robust governance structures contribute significantly to improved financial results. The study highlights the need for commercial banks in Nepal to emphasize increasing external supervision, attempting to improve credit appraisal processes, maintaining adequate capital levels, and strengthening corporate governance mechanisms.

These factors can improve decision-making, risk management and overall performance outcomes. Based on the findings of this research, except credit appraisal measurement all factors like external supervision, capital adequacy levels and corporate governance structures are major determinants of commercial bank performance in Nepal. Accordingly, diverse boards with effective credit appraisal practices, higher capital adequacy levels and robust governance structures contribute significantly to improved financial results.

This study emphasizes the need for commercial banks in Nepal to prioritize increasing external supervision, improving credit appraisal processes, maintaining sufficient capital levels and strengthening corporate governance mechanisms. These factors can help contribute to better decision-making, risk management and performance outcomes in banking sectors in Nepal. The findings from this research hold significant ramifications for stakeholders within this banking industry. Commercial banks should prioritize increasing external supervision by including people from diverse backgrounds, areas of expertise, and perspectives. Diversity within these banks can enhance decision-making processes, spur innovation, and strengthen risk management practices. Commercial banks can increase effectiveness by harnessing diverse experiences and viewpoints from within their ranks.

Banks must also invest in robust credit appraisal processes. This involves employing comprehensive risk evaluation measures and carefully evaluating borrowers for creditworthiness, among other activities. Adoption of these practices helps banks to reduce credit risk while simultaneously improving loan portfolio quality, ultimately leading to greater financial stability and sustainability for themselves. Additionally, banks should maintain sufficient capital levels according to regulatory requirements. Capital adequacy against unexpected losses and strengthens overall banking system resilience and stability. By adhering to regulatory requirements regarding capital levels, banks can ensure adequate protection of their operations and greater stability within the financial sector.

Banks should prioritize strengthening their corporate governance mechanisms, including ensuring board members’ independence, creating effective risk management frameworks and encouraging transparency and accountability. By strengthening corporate governance practices, banks can build investor trust while mitigating agency conflicts and improving overall performance—measures that create a more reliable banking system benefiting them and their stakeholders.

The findings of this research emphasize the significance of key elements, including external supervision, credit appraisal measurement, capital adequacy, and corporate governance within Nepal’s banking sector. Implementing their implications can bring improvement through decision-making, risk management, financial stability and overall performance of financial sector in Nepal.

### 5.1 Academic contributions

This research makes several valuable contributions to the academic literature. Firstly, study provides empirical evidence of the positive relationship between board diversity and financial performance in the unique context of Nepal Commercial Bank. Financial factors such as corporate governance enhances a company’s growth [56], and better leverage to financial stability, present research majorly contributes in the role of corporate governance as an interplay among external supervision, capital appraisal management and capital adequacy for banks performance in Nepal. This contributes to the broader academic discourse on diversity’s impact on decision-making, risk management, and innovation, Gomez [57] also endorsed that diversity is associated with decision-making, risk management, and innovation. Secondly, it reveals the significant role of credit appraisal management practices in enhancing bank performance, emphasizing the importance of robust credit appraisal systems in effective risk management, Kithinji [58]’s study also incorporated that credit appraisal management practices improves profitability of a firm. This adds to the growing body of knowledge on credit risk assessment in the banking sector. Thirdly, we establish a direct link between capital adequacy and organizational performance, demonstrating how maintaining sufficient capital resources support financial stability and growth. This further enriches the understanding of capital adequacy in the context of Nepal. Finally, our findings underscore the pivotal role played by corporate governance structures and mechanisms in shaping the relationships between various factors and organizational performance. This highlights the importance of effective governance, stakeholder alignment, and transparent decision-making practices in the banking sector.

### 5.2 Managerial implications

Our research offers valuable insights for bank management and policymakers. Firstly, it advocates for the active promotion of diversity within the board composition. We suggest that banks should consider diversifying board members in terms of gender, ethnicity, expertise, and backgrounds. Diverse boards are more likely to bring varied perspectives, leading to improved decision-making and innovation. Secondly, we emphasize the continuous improvement of credit appraisal measurement practices. Banks are encouraged to invest in technology, training, and data analytics to enhance their credit risk assessment processes, thereby leading to better risk management and improved performance. Thirdly, we underscore the importance of maintaining an adequate capital base. Banks are recommended to regularly assess their capital adequacy ratios and ensure alignment with regulatory requirements. Adequate capital resources are critical for both supporting growth and enhancing financial stability. Finally, we prioritize corporate governance structures and mechanisms. This includes defining clear roles and responsibilities for board members, ensuring transparency in decision-making and aligning stakeholder interests. Effective governance is foundational to organizational success.

### 5.3 Limitations and future research

This study is not without its limitations. The utilization of cross-sectional data restricts the ability to establish definitive causal relationships. We advise caution when inferring causality from observed associations. Furthermore, the focus on Nepal Commercial Bank may limit the applicability of our findings to other banking systems with unique characteristics. Additionally, potential common method bias in survey responses could impact the accuracy of results. To address these limitations, future research can delve into longitudinal studies to establish the long-term impact of these factors on organizational performance. Comparative research across industries and regions can provide valuable insights into contextual variations. Further research may also focus on exploring the causal relationships between these variables to provide a deeper understanding of the dynamics at play, accounting for external factors such as regulatory changes, macroeconomic conditions, or technological advancements.

In conclusion, this study’s findings provide practical insights for organizations aiming to enhance their performance. By implementing our recommendations, banks can improve financial outcomes and contribute to broader societal goals of diversity, transparency, and sustainable growth. Policymakers and researchers should take heed of these findings to further advance the discourse on governance, risk management, and organizational success. Our research underscores the paramount importance of sound governance practices and prudent financial management, which encompass inclusive decision-making processes, in propelling the success of banks within Nepal’s distinct environment.

## Supporting information

S1 Appendix (DOCX)

S1 Data (CSV)
