# Supplementary material for: The role of corporate governance structures in mediating the relationship between external supervision, credit appraisal measurement, capital adequacy, and performance of commercial banks in Nepal
Source: PLoS One. 2024 Jun 13;19(6):e0303926. doi: 10.1371/journal.pone.0303926 (PMC11175449; doi:10.1371/journal.pone.0303926)
Supplement: S1 Appendix — (DOCX) [file pone.0303926.s001.docx]

**Appendix-1**

|  |  | **SDA** | **DA** | **N** | **AG** | **SAG** |
| --- | --- | --- | --- | --- | --- | --- |
|  | **External supervision[**[**46**](#_ENREF_46)**]** |  |  |  |  |  |
| 1 | The external supervision I received was helpful in enhancing my skills and knowledge. | 1 | 2 | 3 | 4 | 5 |
| 2 | The external supervisor provided constructive feedback that positively influenced my performance. | 1 | 2 | 3 | 4 | 5 |
| 3 | I felt comfortable discussing challenges and seeking guidance from my external supervisor. | 1 | 2 | 3 | 4 | 5 |
| 4 | The external supervision sessions were well-structured and focused on my professional development needs. | 1 | 2 | 3 | 4 | 5 |
| 5 | The external supervisor demonstrated expertise in the areas they were providing supervision for. | 1 | 2 | 3 | 4 | 5 |
| 6 | The external supervisor was approachable and receptive to my questions and concerns. | 1 | 2 | 3 | 4 | 5 |
| 7 | Overall, I am satisfied with the quality of external supervision I received. | 1 | 2 | 3 | 4 | 5 |
|  | **Credit appraisal measurement[**[**47**](#_ENREF_47)**]** |  |  |  |  |  |
| 1 | The financial information provided by the borrower is accurate and reliable. | 1 | 2 | 3 | 4 | 5 |
| 2 | The borrower's credit history demonstrates a consistent record of timely payments. | 1 | 2 | 3 | 4 | 5 |
| 3 | The borrower's debt-to-income ratio is within an acceptable range. | 1 | 2 | 3 | 4 | 5 |
| 4 | The borrower has provided sufficient collateral to secure the credit. | 1 | 2 | 3 | 4 | 5 |
| 5 | The borrower's business plan clearly understands market conditions and potential risks. | 1 | 2 | 3 | 4 | 5 |
| 6 | The borrower has a stable and reliable source of income to repay the credit. | 1 | 2 | 3 | 4 | 5 |
| 7 | The borrower's credit score indicates a low risk of default. | 1 | 2 | 3 | 4 | 5 |
| 8 | The borrower has a positive reputation and track record in previous credit transactions. | 1 | 2 | 3 | 4 | 5 |
| 9 | The borrower has demonstrated a willingness to provide additional information and cooperate during the credit appraisal process. | 1 | 2 | 3 | 4 | 5 |
| 10 | The overall creditworthiness of the borrower is satisfactory. | 1 | 2 | 3 | 4 | 5 |
|  | **Capital adequacy[**[**48**](#_ENREF_48)**]** |  |  |  |  |  |
| 1 | The company maintains an appropriate level of capital to support its operations and absorb potential losses. | 1 | 2 | 3 | 4 | 5 |
| 2 | The company's capital structure is well-balanced and aligns with its risk profile. | 1 | 2 | 3 | 4 | 5 |
| 3 | The company regularly assesses and evaluates its capital needs to ensure they are met adequately. | 1 | 2 | 3 | 4 | 5 |
| 4 | The company complies with regulatory capital requirements applicable to its industry. | 1 | 2 | 3 | 4 | 5 |
| 5 | The company has implemented effective risk management practices to mitigate capital-related risks. | 1 | 2 | 3 | 4 | 5 |
|  | **Performance[**[**49**](#_ENREF_49)**]** |  |  |  |  |  |
| 1 | The company consistently achieves its financial targets and objectives. | 1 | 2 | 3 | 4 | 5 |
| 2 | The company demonstrates steady revenue growth over time. | 1 | 2 | 3 | 4 | 5 |
| 3 | The company maintains a healthy profit margin compared to industry peers. | 1 | 2 | 3 | 4 | 5 |
| 4 | The company effectively manages its operating costs to maximize profitability. | 1 | 2 | 3 | 4 | 5 |
| 5 | The company's return on investment (ROI) is satisfactory. | 1 | 2 | 3 | 4 | 5 |
| 6 | The company consistently generates positive cash flows from its operations. | 1 | 2 | 3 | 4 | 5 |
| 7 | Investors and stakeholders positively perceive the company's financial performance. | 1 | 2 | 3 | 4 | 5 |
| 8 | The company demonstrates efficient utilization of its assets to generate income. | 1 | 2 | 3 | 4 | 5 |
| 9 | The company maintains a strong credit rating and reputation in the market. | 1 | 2 | 3 | 4 | 5 |
| 10 | Overall, the company's financial performance is satisfactory. | 1 | 2 | 3 | 4 | 5 |
|  | **Corporate governance[**[**50**](#_ENREF_50)**]** |  |  |  |  |  |
| 1 | The company has a clear organizational structure that defines the roles and responsibilities of board members, executives, and management. | 1 | 2 | 3 | 4 | 5 |
| 2 | The company has established effective mechanisms to ensure accountability and transparency in decision-making processes. | 1 | 2 | 3 | 4 | 5 |
| 3 | The company's board of directors comprises individuals with diverse backgrounds and expertise. | 1 | 2 | 3 | 4 | 5 |
| 4 | The company has established a code of ethics and conduct that guides the behavior of its directors, executives, and employees. | 1 | 2 | 3 | 4 | 5 |
| 5 | The company maintains a strong system of internal controls to prevent fraud and ensure compliance with laws and regulations. | 1 | 2 | 3 | 4 | 5 |
| 6 | The company has an independent and qualified audit committee overseeing financial reporting and external audits. | 1 | 2 | 3 | 4 | 5 |
| 7 | The company regularly discloses relevant and timely information to shareholders and stakeholders. | 1 | 2 | 3 | 4 | 5 |
| 8 | The company has a process for evaluating and managing conflicts of interest among directors, executives, and related parties. | 1 | 2 | 3 | 4 | 5 |
| 9 | The company promotes a culture of integrity and encourages employees to report any misconduct or unethical behavior. | 1 | 2 | 3 | 4 | 5 |
| 10 | Overall, the company demonstrates a robust corporate governance structure. | 1 | 2 | 3 | 4 | 5 |
